# Meniscal repair results in inferior short-term outcomes compared with meniscal resection: a cohort study of 6398 patients with primary anterior cruciate ligament reconstruction

**DOI:** 10.1007/s00167-017-4793-2

**Published:** 2017-11-13

**Authors:** Eleonor Svantesson, Riccardo Cristiani, Eric Hamrin Senorski, Magnus Forssblad, Kristian Samuelsson, Anders Stålman

**Affiliations:** 1 Department of Orthopaedics, Institute of Clinical Sciences, The Sahlgrenska Academy, University of Gothenburg, Gothenburg, Sweden; 2 Capio Artro Clinic, Valhallavägen, Stockholm, Sweden; 3 Department of Health and Rehabilitation, Institute of Neuroscience and Physiology, The Sahlgrenska Academy, University of Gothenburg, Gothenburg, Sweden; 4 Department of Molecular Medicine and Surgery, Stockholm Sports Trauma Research Center, Karolinska Institutet, Stockholm, Sweden; 5 Department of Orthopaedics, Sahlgrenska University Hospital, Mölndal, Sweden

**Keywords:** Anterior cruciate ligament, ACL, Meniscus, Meniscal repair, Meniscal resection, Reconstruction, Surgery

## Abstract

**Purpose:**

To compare patient reported outcome measures (PROMs) during the first postoperative year between isolated anterior cruciate ligament (ACL) reconstruction and ACL reconstruction with concomitant meniscal intervention.

**Methods:**

Patients who underwent primary ACL reconstruction at Capio Artro Clinic, Stockholm, Sweden, between 1st Jan 2001 and 31st Dec 2014 without concomitant injuries others than meniscal and/or cartilage lesions were included. Five groups of meniscal treatment simultaneously to ACL reconstruction were established; medial meniscal (MM) resection, MM repair, lateral meniscal (LM) resection, LM repair, and MM + LM resection. Patients treated with isolated ACL reconstruction formed a separate group. Preoperative, 6-month and 1-year Knee Injury and Osteoarthritis Outcome Score (KOOS), and Lysholm knee score and Tegner Activity scale were collected. Differences in the change over time were analyzed with an ANOVA for repeated measurements with age at surgery, gender, and cartilage injury as covariates. A univariate ANOVA was applied to analyze PROMs between groups at the final follow-up.

**Results:**

A total of 6398 patients were included (56.8% males, mean age 28.5 ± 10.2 years). The KOOS improved across all subscales for all treatment groups. The mean change over time differed significantly between groups for the subscales symptoms (*p* = 0.017) and activities in daily living (ADL) (*p* < 0.001). Symptoms was least improved in the MM repair group, while the MM + LM resection group showed the largest improvement. For the ADL subscale, the isolated ACL reconstruction group showed the least improvement and the MM + LM resection group showed the major improvement. At 1-year follow-up, a significant difference between the groups was found for the subscale symptoms (*p* = 0.019), where the MM repair group reported the lowest score [mean 78.4 (95% CI 76.3–80.5)]. No significant differences were found between groups in change of the Lysholm score over time; however, at 6 months, the difference between groups was significant (*p* = 0.006) with the meniscal repair groups reporting the lowest scores.

**Conclusion:**

Patients with concomitant meniscal resection are able to reach the same subjective knee function as isolated ACL reconstructions as early as 6 months postoperatively. However, patients with meniscal repair may have slightly worse subjective knee function at both 6- and 12-month follow-up. These findings could help clinicians to set realistic short-term expectations for patients undergoing ACL reconstruction with simultaneous meniscal intervention.

**Level of evidence:**

3.

## Introduction

The risk for simultaneous injury to the menisci in the occurrence of an anterior cruciate ligament (ACL) injury is substantial [1, 2]. The menisci are important structures for load transmission and are fundamental for preserved knee-health. Deficiency of the menisci can result in increased knee laxity [3–5] which in turn may lead to higher stress forces on the ACL [6] and perhaps abnormal cartilage load. In a long-term perspective, patients suffering from combined ACL and meniscal tears have been shown to present with a higher prevalence of osteoarthritis (OA) compared with patients with an isolated ACL injury [7, 8]. Therefore, thorough consideration regarding appropriate treatment for the meniscus is important in order to preserve its function and prevent the development of OA.

Long-term (5–10 years) comparisons of treatment strategies for meniscal tears have concluded that meniscal resection results in a higher postoperative rate of radiographic OA compared to meniscal repair [9–12]. Although the radiographic findings of OA are not always accompanied by symptomatic OA [13, 14], a resection of the meniscus seems to result in worse patient reported outcome compared to repair at long-term follow-up [11, 12]. However, a recent study highlighted that these long-term effects of meniscal resection may not exist in the short-term [15]. At 2-year follow-up, patients who had undergone any of; medial meniscal (MM) resection, lateral meniscal (LM) resection or LM repair in addition to ACL reconstruction reported similar Knee injury and Osteoarthritis Outcome Score (KOOS) as patients receiving an isolated ACL reconstruction. In contrary to previous long-term studies, a simultaneous MM repair was shown to result in significantly lower scores. This study also revealed that all meniscal treatment groups, except the LM resection, reported significantly lower preoperative scores compared to isolated ACL reconstruction and that these differences were equalized for all treatment groups except for the MM repair group, at 2-year follow-up [15]. Knowledge about the short-term effects on subjective knee function after concurrent meniscal resection or repair in ACL reconstruction is important in clinical practice. Patients with combined meniscal injuries may present worse symptoms preoperatively, but with correct treatment and rehabilitation following surgery they have large potential to improve and “catch up” with patients of an isolated ACL injury. The purpose of this study was to compare patient reported outcome measures (PROMs) during the first postoperative year between isolated ACL reconstruction and combined ACL reconstruction and meniscal intervention in order to increase the awareness of the outcome during the time of rehabilitation. It was hypothesized that presence of meniscal injury would influence preoperative PROMs negatively; however, the short-term outcome would be comparable to patients with an isolated ACL reconstruction.

## Materials and methods

This retrospective cohort study was conducted at Capio Artro Clinic, Sophiahemmet, Stockholm, Sweden. Patients aged 13 years or older who underwent primary ACL reconstruction using bone-patellar-tendon-bone (BPTB) or hamstring tendon (HT) autografts during the study period 1st Jan 2001 to 31st Dec 2014 and did not present with any concomitant injuries other than meniscal and/or cartilage lesions were considered eligible. The study period was chosen since registration of meniscal injuries in the local registry started in 2001, and, in order to get a proper 1-year follow-up, patients undergoing surgery later than 31st Dec 2014 could not be included. Patients with contralateral or revision ACL reconstruction were excluded, as well as patients with untreated meniscal injury and patients receiving more than one type of meniscal treatment such as combinations of repair and resection. However, combined MM + LM resections were included and analyzed as a separate treatment group.

### Surgical technique and rehabilitation

For the ACL reconstructions performed with HT graft, the semitendinosus was primarily harvested and prepared as a triple or a quadruple graft. If the graft width was insufficient (less than 8 mm), the gracilis tendon was additionally harvested and combined with the semitendinosus graft. The graft was fixed with an Endobutton fixation device (Smith & Nephew, Andover, Mass, USA) on the femoral side and Ultrabraid (Smith & Nephew, Andover, Mass, USA) or Ethibond no. 2 sutures (Ethicon Inc, USA) tied over an AO bicortical screw with a washer on the tibial side. For patients undergoing reconstruction with BPTB graft, the central third of the patellar tendon was harvested with two bone blocks. The graft was fixed with Endobutton fixation device (Smith & Nephew, Andover, Mass, USA) on the femoral side and with an interference screw on the tibial side (Softsilk, Smith and Nephew, Andover, Mass, USA). Meniscal repair was performed with an all-inside arthroscopic technique using FasT-Fix suture anchor device (Smith and Nephew, Andover, Mass, USA) or inside-out technique in the dorsal or middle thirds of the meniscus, and with outside-in technique in the anterior third of the meniscus.

All patients were recommended the same rehabilitation protocol. In case of isolated ACL reconstruction or ACL reconstruction with simultaneous meniscus resection, full weight bearing was recommended as tolerated and the early rehabilitation phase emphasized on regained range of motion (ROM), reduction of swelling, and gait correction. A carefully progressive program with increased strengthening and proprioceptive exercises followed. Rehabilitation was restricted to closed chain exercises during the first 3 months. Patients treated with meniscal repair wore a hinged knee brace for 6 weeks. Flexion was limited from 0° to 30° the first 2 weeks, 0°–60° week 3–4, and 0°–90° week 5 and 6. Starting from the 7th week, the brace was discontinued and progressive weight bearing was allowed. Return to sports was recommended at earliest 6 months postoperatively depending on type of sport and knee function.

### Outcome

Outcome measurements in this study were the Knee injury and Osteoarthritis Outcome Score (KOOS) [16, 17], the Lysholm knee score [18] and the Tegner activity scale [18, 19]. The KOOS has been validated to determine subjective outcome in patients with knee injuries and knee osteoarthritis. It is divided into five subscales—pain, symptoms, activities in daily living (ADL), function in sport and recreation, and knee-related quality of life (QoL). A score of 0 represents the worst possible outcome while 100 is the maximum score for a subscale. The Lysholm score comprises eight condition-specific domains which are summarized as a score ranging from 0 (worst) to 100 (best). The Tegner score ranges from 0 to 10, with 0 indicating severe disability and 10 indicating participation in competitive knee-demanding sports at elite level. Preoperative data were obtained for all outcomes. The Lysholm and Tegner scores were evaluated at 6 months follow-up, and the KOOS at 1-year follow-up. Only patients with available data at both the aforementioned follow-ups as well as preoperatively were included in analyzes of changes over time. For the KOOS, the number of patients with available data differed between the five subscales; hence, the study sample for each subscale was slightly different. Any missing data was addressed according to KOOS user guide 1.1 [20].

### Data sources and measurement

All ACL reconstructions performed at Capio Artro Clinic, Stockholm are registered in the clinic’s local registry. The registry is divided into separate parts for the surgeon and the patient. Regarding the surgeon’s part, data are reported prospectively into the database immediately following surgery. Compliance is 100% since the system automatically requires accurate reporting of the surgical data by the surgeon to be able to proceed to the patient’s medical record. Details regarding the surgery such as graft choice, fixation methods, concomitant injuries, and all interventions and procedures to the knee are reported. Information about date of surgery, length of surgery, and any previous surgeries to the ipsilateral or contralateral knee are also recorded.

Prior to surgery, patients are asked to complete surveys including KOOS, Lysholm and Tegner, and the results are transferred into the database by hospital employees. All patients are offered a follow-up appointment with a physiotherapist 6 months after surgery during which the Lysholm and Tegner questionnaires are completed again. The KOOS questionnaire for 1-year follow-up is distributed via the Swedish national knee ligament register (SNKLR).

This study was conducted according to the WMA Declaration of Helsinki. Investigators had only access to unidentified data and the study was approved by the Regional Ethics Committee, Karolinska Institutet (Diarienumber 2016/1613-31/2).

### Statistical analysis

All data were analyzed using IBM SPSS Statistics (Version 23.0, IBM Corp, Armonk, New York, USA). Tables and diagrams were generated using Microsoft Excel for Windows (Version 14.0.7, Microsoft Corp, Redmond, Washington, USA). Demographic and clinical data as well as PROMs were summarized with standard descriptive statistics, such as frequency, mean, and standard deviation. Differences between groups in baseline characteristics were analyzed. For the variable age, one-way ANOVA followed by post hoc Tukey HSD test was performed. The Chi square test was used for the variables gender and concomitant cartilage lesion. Differences in change over time were analyzed with an ANOVA for repeated measurements (Group * Time) with age at surgery, gender, and cartilage injury as covariates. In addition, an analysis of difference between groups at the final follow-up for each outcome was analyzed by applying a univariate ANOVA with age at surgery, gender, and cartilage injury as covariates. The applied covariates were chosen since several previous register studies from Scandinavia have indicated that these factors could influence PROMs after ACL reconstruction [21–24]. A significance level of 5 percent (two-tailed) was used.

## Results

**Fig. 1:**
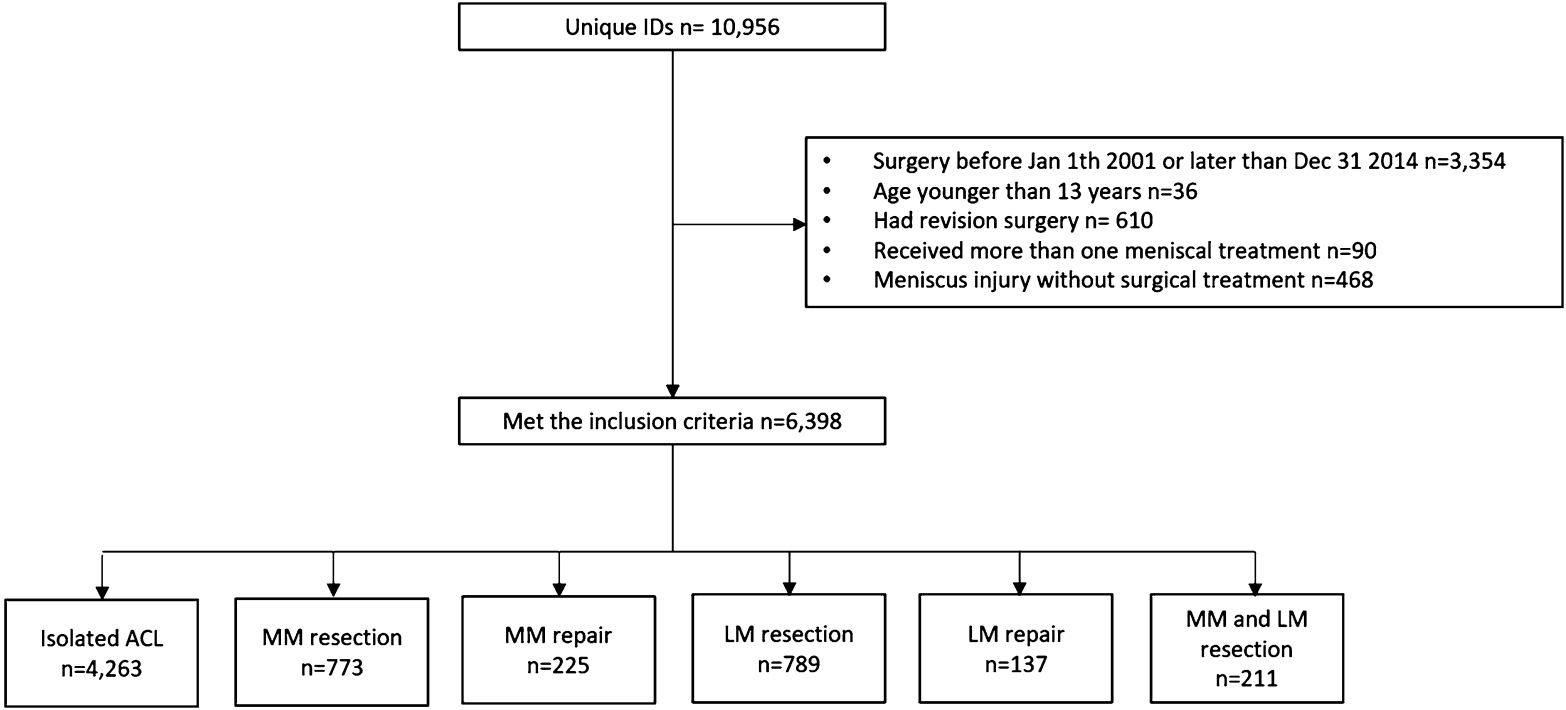
Flow-chart of patient-inclusion process. *ACL* anterior cruciate ligament, *MM* medial meniscus, *LM* lateral meniscus

A total of 10,956 patients were reviewed for eligibility in the local database, of which 6,398 met inclusion criteria. The study population consisted of 56.8% males with a mean age of 28.5 ± 10.2 years. Six groups were generated depending on meniscal treatment; Isolated ACL reconstruction, MM resection, MM repair, LM resection, LM repair, and combined MM + LM resection (Fig. 1). In two treatment groups, the proportions of gender differed significantly (*p* < 0.001). The MM repair group had significantly more women (53.8%) while the combined MM + LM resection group consisted of significantly fewer women (35.1%). Further, there was a significant difference in age among the treatment groups (*p* < 0.001). Patients in the LM repair group were the youngest, while the oldest mean age was found for the MM + LM resection and the MM resection groups. The demographic data are presented in detail in Table 1.

**Table 1:** Demographic data for study population

	Isolated ACL	MM resection	MM repair	LM resection	LM repair	MM + LM resection	Total cohort
Number (%)	4263 (66.6)	773 (12.1)	225 (3.5)	789 (12.3)	137 (2.1)	211 (3.3)	6398 (100.0)
Age at surgery (mean ± SD)	28.3 ± 10.0	32.5 ± 10.5	24.5 ± 8.9	27.2 ± 9.4	22.2 ± 9.4	31.8 ± 11.4	28.5 ± 10.2
Gender, no. (%)							
Male	2319 (54.5)	483 (62.5)	104 (46.2)	513 (65.0)	78 (56.9)	137 (64.9)	3634 (56.8)
Female	1944 (45.6)	290 (37.5)	121 (53.8)	276 (35.0)	59 (43.1)	74 (35.1)	2764 (43.2)
Chondral lesion, no. (%)	612 (14.4)	217 (28.1)	57 (25.3)	178 (22.6)	32 (23.4)	69 (32.7)	1165 (18.2)
Time from injury to surgery							
Number of patients with available data	3474	633	214	680	134	169	5304
Days (mean ± SD)	449 ± 825	795 ± 1220	441 ± 918	413 ± 747	389 ± 829	738 ± 1089	493 ± 893
Baseline Tegner activity score							
Number of patients with available data	3005	588	190	605	115	156	4659
Tegner score, median (range)	7 (0–10)	7 (1–10)	7 (1–10)	8 (0–10)	8 (1–10)	7 (0–10)	7 (0–10)

*ACL* anterior cruciate ligament, *MM* medial meniscus, *LM* lateral meniscus, *SD* standard deviation, *No* number

### Knee injury and osteoarthritis outcome score

**Fig. 2:**
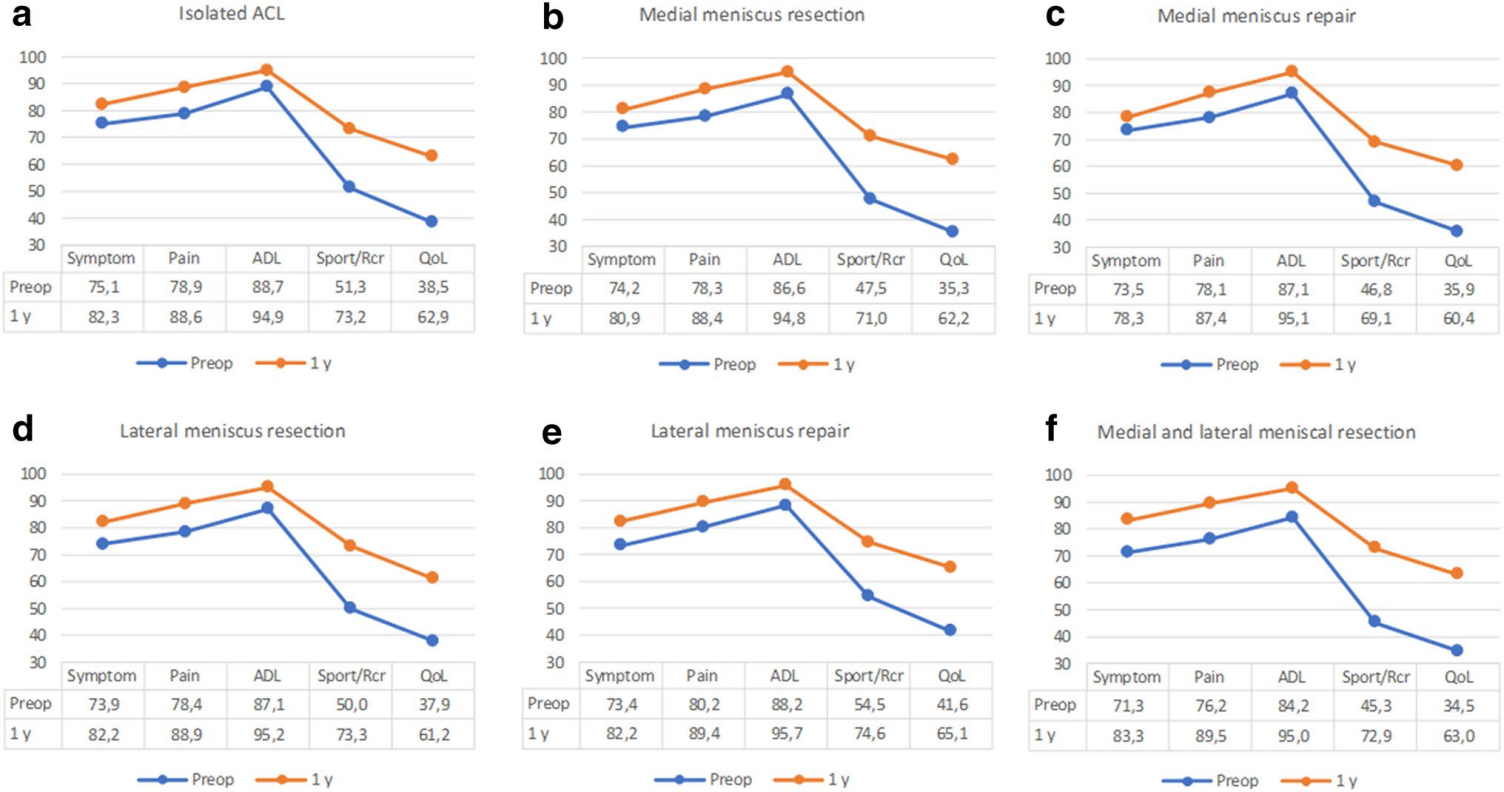
**a**–**f** Unadjusted preoperative and 1-year Knee Injury Osteoarthritis Outcome Score displayed for each treatment group, respectively. Note that the scale on the y-axis starts at a score of 30. *Preop* preoperative, *y* year, *ACL* anterior cruciate ligament, *ADL* activities in daily living, *Sport*/*Rcr* sports and recreation, *QoL* quality of life

The proportion of patients with available KOOS data both preoperatively and at 1 year for each subscale was as follows: symptoms 87.4%, pain 86.3%, ADL 86.1%, sport and recreation 81.8%, QoL 84.0%. Throughout all treatment groups, the lowest preoperative KOOS scores were found in the subscales sport and recreation and QoL (Fig. 2 a–f). The KOOS improved for all treatment groups across all subscales during the 1-year follow-up (Fig. 2a–f). For the subscales symptoms and ADL, the mean change over time differed significantly between the groups (*p* = 0.017 and *p* < 0.001, respectively). Symptoms was least improved in the MM repair group while the MM + LM resection group showed the largest improvement. For the ADL, the isolated ACL reconstruction group showed the smallest improvement, whereas the MM + LM resection group showed the largest (Table 2). At 1-year follow-up, a significant difference between the groups was found only for the subscale symptoms (*p* = 0.019). The lowest score in symptoms was found in the MM repair group [mean 78.4 (95% CI 76.3–80.5)] while the mean score of the other groups ranged from 81.5 to 83.6 (analysis adjusted for age, gender, and cartilage).

**Table 2:** Mean change from preoperative score to 1-year follow-up presented for each KOOS subscale

	Isolated ACL (*n* = 3486–3727)	MM resection (*n* = 646–688)	MM repair (*n* = 186–196)	LM resection (*n* = 647–695)	LM repair (*n* = 104–113)	MM + LM resection (*n* = 162–176)	*p* value
Symptom	7.1	6.9	4.6	8.1	8.4	12.2	0.017
Pain	9.6	10.7	8.6	10.5	8.5	13.9	n.s
ADL	6.2	8.2	8.0	8.2	7.7	10.7	< 0.001
Sport/Rcr	21.9	23.7	21.9	23.3	19.9	27.7	n.s
QoL	24.5	26.6	24.6	23.6	24.0	28.3	n.s

Covariates applied to the model are age, gender and cartilage injury

*ACL* anterior cruciate ligament, *MM* medial meniscus, *LM* lateral meniscus, *ADL* activities in daily living, *Sport/Rcr* sports and recreation, *QoL* quality of life, *n.s* non-significant

### Lysholm score

In total, 83.9% of the study population had valid data on the Lysholm knee score both preoperatively and at 6 months follow-up. All treatment groups improved and no difference in improvement between the groups was observed (*p* = 0.113) (Table 3). In the additional analysis of solely, the Lysholm score at 6 months, the Lysholm score differed significantly between the groups at this time point (*p* = 0.006). The LM and the MM repair groups reported the lowest mean scores (68.2 and 71.7 points, respectively), while the other groups reported mean scores ranging from 75.2 to 76.8 points.

**Table 3:** Mean Lysholm score at baseline, at 6 months postoperatively, and the mean change between these timepoints for each treatment group, respectively

	Isolated ACL *n* = 3595	MM resection *n* = 663	MM repair *n* = 179	LM resection *n* = 660	LM repair *n* = 107	MM + LM resection *n* = 166	*p* value
	Mean ± SD	Mean ± SD	Mean ± SD	Mean ± SD	Mean ± SD	Mean ± SD	
Baseline	68.2 ± 20.7	63.7 ± 21.6	65.2 ± 24.0	67.3 ± 20.9	64.8 ± 24.8	62.2 ± 20.9	n.s
6 months	76.9 ± 27.2	74.9 ± 28.3	72.4 ± 29.5	77.3 ± 26.5	70.0 ± 34.3	74.0 ± 30.3	
Change^a^	8.7	11.4	6.8	9.8	4.6	11.9	

*ACL* anterior cruciate ligament, *MM* medial meniscus, *LM* lateral meniscus, *SD* standard deviation, *n.s* non-significant

^a^Covariates applied to the model for change over time (preoperative to 6 months postoperatively) are age, gender, and cartilage injury

### Tegner activity score

At 6 months, the Tegner activity score had decreased from the pre-injury score throughout all treatments groups. No significant differences were found between the groups (data not shown).

## Discussion

The most important finding of the present study was that patients treated with meniscal repair report inferior short-term PROMs compared with meniscal resection in the setting of primary ACL reconstruction. Medial or lateral meniscal repair was shown to result in small but significantly inferior results in the Lysholm score at 6 months postoperatively, and the medial repair group also reported a significantly lower score in the KOOS subscale symptoms at 1-year follow-up. Nevertheless, patients with concomitant meniscal injuries improve considerably over the first postoperative year and are generally able to reach the same subjective knee function as an isolated ACL injury.

A similar analysis to the present study with 2-year follow-up from the Norwegian knee ligament registry showed that patients treated with MM repair in addition to ACL reconstruction had significantly lower scores in the KOOS subscales symptom and QoL compared with isolated ACL reconstruction, a finding not seen for any other meniscal treatment group [15]. This study confirms these findings, since the only difference between the groups was seen in the KOOS symptoms subscale, where the MM repair group reported significantly lower scores. The current study investigated the 1-year KOOS score, however, a recent study reported that the 1-year KOOS is equivalent to the 2-year score for patients with and without concomitant meniscal injury [25]. Thus, these findings are likely representative for the first 2 years after surgery. Furthermore, this study showed that significant differences exist with regard to the change in the first year improvement of KOOS, especially for the combined MM + LM resection group. This group was found to improve significantly more in the subscales symptoms and ADL compared to the other groups, suggesting that these patients may be more symptomatic preoperatively but are treated effectively with meniscal resection and can achieve similar KOOS in the short-term perspective as compared with other treatment groups.

The Lysholm score was evaluated 6 months postoperatively and adds knowledge with regard to the subjective outcome during rehabilitation. Although no differences were seen in change over time from the preoperative to the postoperative score between the groups, a tendency towards a slightly inferior improvement was seen in the LM repair group and, at 6-month follow-up, both meniscal repair groups reported lower scores. The postoperative management of meniscal repairs is more restraint compared with both isolated ACL reconstructions and/or meniscal resections due to concerns that weight bearing and knee flexion may cause gapping of the meniscus and generate intolerable tension and hoop stress [26, 27]. However, controversies exist since previous studies have reported similar results between restricted and accelerated rehabilitation following meniscal repair, implicating safety of a more aggressive accelerated rehab [27–30]. Biomechanical studies have shown that unrestricted ROM do not place undue stress on meniscal repairs [31] and simulated gait may actually produce compression, not gapping, of some meniscal tear types [32]. In fact, it has been suggested that functional stresses applied to the meniscus when implementing an accelerated rehabilitation protocol may promote healing of the repair [33] and instead, it has been emphasized that early unlimited ROM is important to avoid complications following a concomitant ligamentous procedure [31, 32]. The current study shows that even though a restricted and slow rehab is chosen after meniscal repair, the patients will reach a fair, but slightly lower Lysholm score compared with isolated ACL reconstruction, as early as 6 months after surgery. Based on the KOOS at 1-year follow-up, the subjective function is clinically similar among all groups, regardless of meniscal treatment.

Most importantly, this study confirms that meniscal resection is an effective procedure in the short-term. However, this finding particularly highlights the importance of further care of these patients in order to prevent OA, since long-term studies are conclusive regarding the increased risk of OA following meniscal resection [7, 9, 11, 12, 34]. A well-functioning knee in the short-term after meniscal resection increases the possibilities for the patients to participate in sports with the risk of high loads to the knee and subsequent OA. Therefore, clinicians should inform patients about their prognosis to enable this patient population to make wise future choices for preserved knee-health. Continued follow-up of this population is desirable in order to counter the development of OA at an early stage, as well as for research-related purposes and gained insight. Furthermore, preservation of the menisci is advocated whenever possible. This is particularly applied to the younger population, which is in concordance with the results of this study since the resection groups consisted of significantly older patients [35, 36].

This study is strengthened by the large study population. Moreover, all patients received the same rehabilitation recommendations and the surgery was standardized. These characteristics make this study different from previous studies on national registries which include patients operated at different clinics with different surgical techniques and rehabilitation protocols. The main limitation with this study is that although the patients were grouped according to meniscal treatment, nothing is known about the characteristics of the lesion. Neither is the proportion of meniscal removal in the resection groups known since the registry does not contain this information. The indications for choice of meniscal treatment are unknown with the inevitable consequence of possible selection bias. Furthermore, it should be emphasized that the outcome measures in this study may not be capable of discriminating subjective differences that are clinically important. The KOOS has been frequently used to study outcome after ACL reconstruction; however, differences in outcome reported by previous studies are many times less than the minimal detectable change [25, 37]. The KOOS as an outcome measure may therefore be difficult to interpret and the findings of this study should be treated with caution, keeping the intrinsic limitations associated with PROMs in mind. Nevertheless, it is important for clinicians to have knowledge of the patients’ perspective on knee function in order to individualize the rehabilitative care and to inform the patients and set realistic expectations regarding the short-term prognosis after ACL reconstruction with concomitant meniscal treatment.

## Conclusion

Patients with concomitant meniscal resection are able to reach the same subjective knee function as isolated ACL reconstructions as early as 6 months postoperatively. However, patients with meniscal repair may have slightly worse subjective knee function at both 6- and 12-month follow-up. These findings could help clinicians to set realistic short-term expectations for patients undergoing ACL reconstruction with simultaneous meniscal intervention.
